# Molecular Insights into the Heme‐Binding Potential of Plant NCR247‐Derived Peptides

**DOI:** 10.1002/cbic.202400920

**Published:** 2025-01-20

**Authors:** Sonali M. Vaidya, Dhruv C. Rathod, Anuradha Ramoji, Ute Neugebauer, Diana Imhof

**Affiliations:** ^1^ Department: Pharmaceutical Biochemistry and Bioanalytics Institution: Pharmaceutical Institute University of Bonn, Address 1: An der Immenburg 4 Bonn Germany; ^2^ Department: Leibniz Institute of Photonic Technology Institution: Member of Leibniz Health Technologies Member of the Leibniz Centre for Photonics in Infection Research (LPI), Address 2 Jena Germany.; ^3^ Department: Institute of Physical Chemistry (IPC) and Abbe Center of Photonics (ACP) Institution: Member of the Leibniz Centre for Photonics in Infection Research (LPI), Friedrich-Schiller-University Jena, Address 3 Jena Germany; ^4^ Department: Center for Sepsis Control and Care Institution: Jena University Hospital, Address 4 Jena Germany

**Keywords:** Heme-binding, NCR247 peptides, Cystein-rich peptides, Disulfide-bridged, Bioanalytical characterization.

## Abstract

Heme is involved in many critical processes in pathogenic bacteria as iron acquisition by these microorganisms is achieved by either direct uptake of heme or use of heme‐binding proteins called hemophores. Exploring the underlying mechanisms on a molecular level can open new avenues in understanding the host‐pathogen interactions. Any imbalance of the heme concentration has a direct impact on the bacterial growth and survival. Thus, heme‐regulated proteins that are involved in heme homeostasis poise to be promising targets for research. Similarly, naturally occurring compounds, including cysteine‐rich peptides from either plant secondary metabolites or venom toxins from vertebrates and invertebrates, have been studied for their therapeutic potential. NCR247 is such a cysteine‐rich peptide, known to be crucial for nitrogenase activity in *M. truncatula* and its symbiotic relation with *S. meliloti*. NCR247‐derived peptides were suggested to serve as high‐affinity heme‐binding molecules with remarkable heme‐capturing properties. A comprehensive biochemical and computational analysis of NCR247‐derived peptides, however, redefines their heme‐binding capacity and consequently their potential therapeutic role.

## Graphical abstract

Heme‐binding to Cys‐rich, plant‐derived NCR247 peptides was investigated using biochemical and bioanalytical approaches including UV/Vis and rRaman spectroscopy. The interaction of these disulfide‐bonded peptides plays a critical role in the binding mode with heme. The observations were further substantiated by *in silico* studies. This investigation redefines perceptions of the heme‐binding capacity of NCR247 peptides.

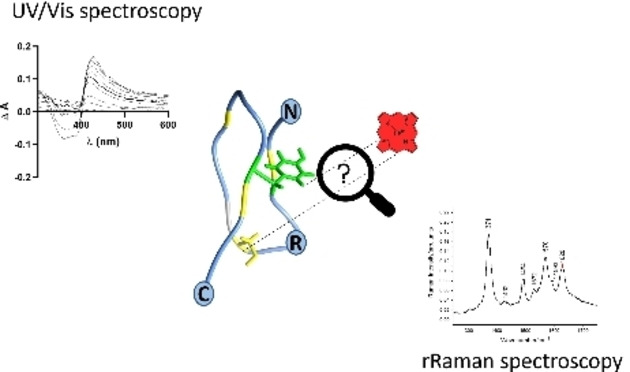

## Introduction

Iron is a crucial metal cofactor for a variety of bacterial processes indispensable for colonization, growth, and reproduction inside a host.[^1^, ^2^, ^3^, ^4^] The strategies for iron acquisition are multifaceted, but in the majority of cases depend on a sophisticated bacterial heme‐uptake machinery. In particular, pathogenic bacteria have developed distinct pathways for host heme sequestration, transmembrane heme transport, and heme degradation for removal of the central iron ion.^[1]^ Such heme‐utilization systems and the proteins involved in the bacterial cellular heme‐uptake machinery have been the focus of numerous investigations in the past.^[5]^ Many of these bacterial proteins are known to bind heme transiently with modest to high affinity, i. e., K_D_ values are in the range of high nM to low μM,^[6]^ which can be exemplified with hemophore‐like protein HmuY from *Porphyromonas gingivalis*, iron‐regulated surface determinant (Isd) proteins from *Staphylococcus aureus*, and iron response regulator (Irr) protein from *Rhizobium leguminosarum*.^[5]^

The majority of heme in humans is found in hemoglobin (Hb), which, at the same time, represents one of the major sources of iron for pathogenic bacteria.[^5^, ^7^] The heterotetramer hemoglobin is transformed into methemoglobin (MetHb) and captured by the serum protein haptoglobin (Hp) as an α/β heterodimer, rendering both, MetHb and MetHb−Hp, the most important sources for bacterial heme acquisition,^[1]^ apart from other heme‐carrying proteins such as myoglobin and the heme‐scavenger hemopexin.[^8^, ^9^, ^10^]

In 2022, Sankari *et al*. reported a putative heme‐sequestering role of the plant‐derived peptide NCR (nodule‐specific cysteine‐rich) 247, a 24mer cationic peptide with four cysteines.^[11]^ Such peptides, produced in the root nodules of e. g., *Medicago truncatula* and *Medicago sativa* (alfalfa) owing to their symbiotic relationship with the Rhizobium bacterium *Sinorhizobium meliloti*, have been extensively studied in the past as valuable secondary metabolites with therapeutic potential^[12]^. The symbiotic cells in the root nodules harbour an ample source of biologically active and antimicrobial compounds that are yet to be characterized. Different organisms including prokaryotes as well as eukaryotes use especially cysteine‐rich peptides (CRPs) as signalling or defence molecules.[^13^, ^14^, ^15^, ^16^, ^17^] *M. truncatula* contains >700 specific cysteine‐rich peptides of varying lengths that are expressed in its nodules and promote endocytosis of bacteria. The endocytosis within the membrane components and cytoplasm of the cells of the root nodules contributes to the formation of nitrogen‐fixing bacteroid.^[18]^

The NCR peptides are known to exclusively contain four or six cysteine residues, usually at conserved positions.[^12^, ^14^] These peptides can be of cationic, neutral and/or anionic nature and have a plethora of bacterial targets and modes of action contributing to their antimicrobial properties.^[19]^ Among all the NCR peptides investigated so far, NCR247 has been studied extensively for its strong antimicrobial activity against pathogenic bacteria including *Enterococcus faecalis*, *Staphylococcus aureus*, *Klebsiella pneumoniae*, *Acinetobacter baumannii*, *Pseudomonas aeruginosa*, and *Escherichia coli*.[^19^, ^20^]

At lower concentrations, this peptide is known to affect the transcription, translation, and cell growth of the rhizobium *S. meliloti* and has antimicrobial or bactericidal effects at higher concentrations.[^18^, ^21^, ^22^] Studies have also shown the effect of disulfide cross‐linking in the NCR247 peptide on the symbiotic relationship between the host and the microbe (*M. truncatula* and *S. meliloti*), its effect on nitrogen fixation as well as in altering physiological functions owing to host innate immune response.^[23]^ Additionally, other reports also discussed the impact of synthetic derivatives and mutants of this peptide in altering the antimicrobial properties of the parent compound.^[20]^

According to the studies of Sankari *et al*.,^[11]^ the disulfide‐bonded NCR247 peptide binds heme with a rather high affinity (nM range) if involved in a structural hexamer comprised of six NCR247 and six heme molecules. Based on the assumptions of the study, this recruitment is supposed to be necessary because the heme iron is needed for the nitrogenase activity and thereby fixes the available nitrogen for the plant. Although the peptide contains a classical heme‐binding CP motif,^[24]^ its cysteines are all engaged in disulfide bridges. Therefore, to understand the heme‐binding potential and the interactions of the 24mer disulfide‐bonded peptide on the molecular level, we were interested to reconnoiter the binding interaction of different variants and disulfide‐bonded isomers of NCR247 using bioanalytical and spectroscopic techniques including UV/vis spectroscopy, circular dichroism (CD) spectroscopy, resonance Raman (rRaman) spectroscopy as well as *in silico* studies to comprehend and precisely define the interaction of heme with NCR247 and its derivatives. Our results show that the heme‐binding potential of NCR247 peptides is limited by the existing disulfide bonds and the binding capacity of Tyr20 is rather weak. The *in silico* analysis confirming the experimental results allow to provide a rationale for a heme‐binding behaviour of the NCR247 peptide and its derivatives different from the earlier reported.

## Results and Discussion

### NCR247‐Derived Peptides Possessing Different Disulfide Connectivities

**Figure 1:**
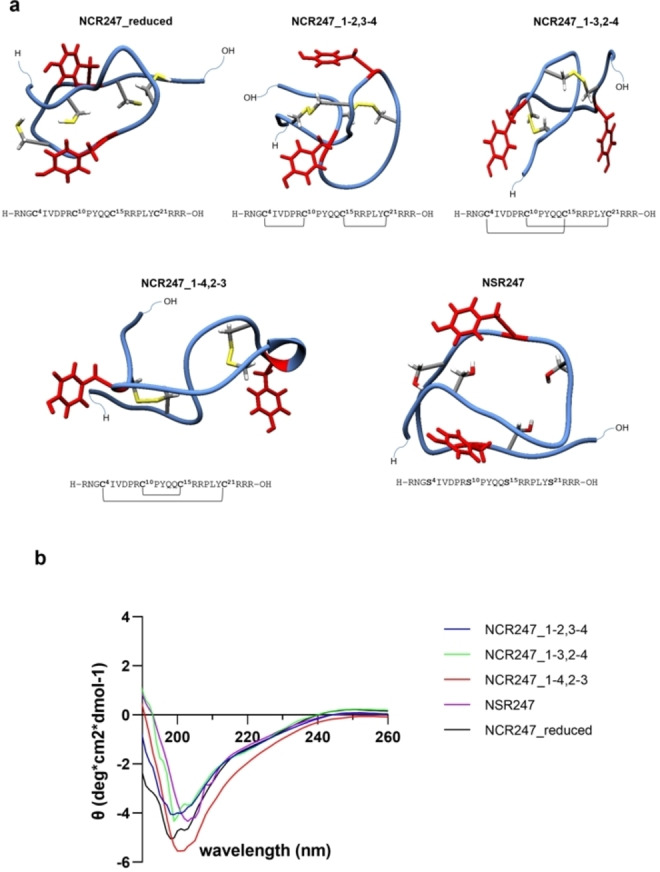
Sequence and structural features of NCR247‐derived peptides. a, C−I‐TASSER modelled 24mer NCR247 and derived peptides illustrating the disulfide connectivities in the disulfide‐bonded isomers with the Cys residues marked in yellow and Tyr residues in red. b, Circular Dichroism (CD) spectra of the 24mer NCR247 peptides and mutant NSR247. The spectra indicate a highly flexible nature of the peptides representing a random coil structure.

**Figure 2:**
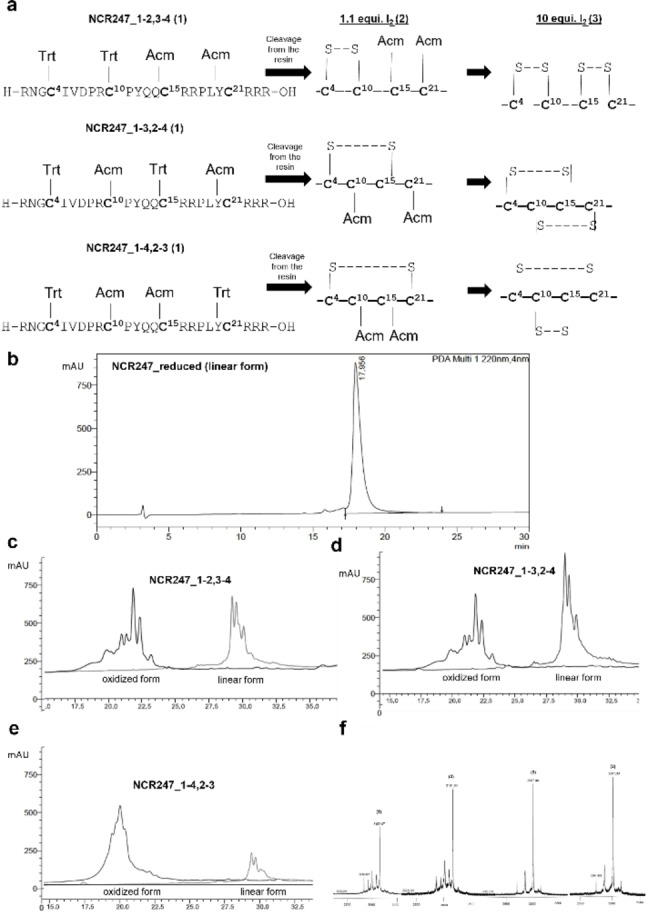
Protecting groups scheme for synthesis and assembly of NCR247 peptides and derivatives. a, Illustration of NCR247 isomers during synthesis, with specific Cys residues protected with Trt and Acm groups, followed by cleavage from the resin (using reagent K and 95 % TFA), addition of first (1.1 equivalence of I2) and second oxidation (10 equivalence of I2). b, RP‐HPLC profile of NCR247_reduced form (linear) with retention time at 29.72 min, analysed on a Knauer C18 column using a gradient elution method of 0–60 % MeCN+0.1 %TFA in 60 min. c,d,e, RP‐HPLC profile of NCR247 derivatives during oxidation from reduced linear form to oxidized form with closed disulfide bridges. The overlay indicates the linear NCR247 derivatives and the oxidized forms. The shift in the retention time indicates the disulfide bridge formation, which was also confirmed with f, MALDI‐MS analysis using carbamidomethylation (CAM).

To clarify the disulfide connectivity existing in NCR247 (Figure 1a) and its properties concerning heme binding, all three possible 2‐disulfide‐bonded isomers were synthesized by solid‐phase peptide synthesis (SPPS) using the Fmoc‐strategy and the incorporation of the protected cysteine pairs as either Cys(Acm) or Cys(Trt). The following linkages were produced in the NCR247 variants: 1) isomer with bridges between Cys4‐Cys10 and Cys15‐Cys21 (NCR_1–2,3–4, numbering according to the order of the cysteines in the sequence), 2) isomer with bridges Cys4‐Cys15 and Cys10‐Cys21 (NCR_1–3,2–4), and 3) isomer with bridges Cys4‐Cys21 and Cys10‐Cys15 (NCR_1–4,2–3). In addition, the mutant (NSR247) with all cysteine residues replaced with serine was synthesized to include a non‐bridged Cys‐mimicking analogue as negative control in the study (Table S1). The disulfide bridge formation in the three disulfide‐isomers was achieved by stepwise oxidation in solution in a one‐pot reaction using iodine as oxidizing agent (Figure 2) according to an earlier described protocol (Supplementary Information, Methods).[^25^, ^26^]

### Conformational Studies of NCR247 Peptides

In order to explore the conformation and to gain a deeper insight into the secondary structure of NCR247 and its derivatives, circular dichroism (CD) spectroscopy analysis in neutral buffer was performed. The experimental set‐up was adapted from earlier research[^27^, ^28^, ^29^]. The 24mer parent peptide revealed high flexibility with low CD signals (θ <1) as can be seen in Figure 1b. The CD‐spectra of all the oxidized NCR247 peptides as well as the reduced form and Ser mutant exhibited a flexible random coil structure.

### NCR247‐Derived Peptides Bind Heme in Moderate To Low μm Range

**Figure 3:**
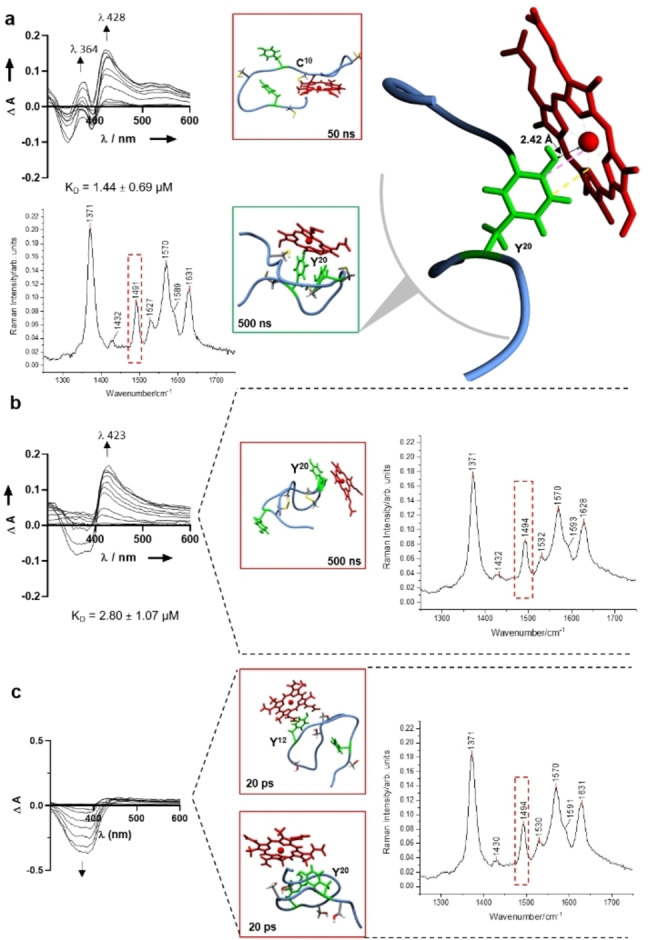
Heme‐binding analysis of NCR247 peptides. a, UV/vis spectroscopic analysis upon addition of increasing concentrations of heme (0.4–40 μM) to NCR247_reduced (on top left), *in silico* docking analysis of the peptide with heme (middle) highlighting residues involved in heme binding, a zoomed‐in illustration (on the right) showing the heme‐coordination to Tyr20 with π‐π interactions depicted in yellow and cation‐π interactions in pink. The rRaman spectrum of the respective heme‐peptide (1 : 1) complex is shown on bottom left. b and c, UV/vis spectroscopic analysis upon addition of increasing concentrations of heme (0.4–40 μM) to NCR247 peptides and analogues (on the left: b, NCR247_1–4,2–3. c, NSR247), *in silico* docking analysis of the respective peptide with heme (middle) showing residues involved in heme binding and rRaman spectra of the respective heme‐peptide (1 : 1) complex (on the right). The complexes shown in the green box are stable for the stated amount of time (s), while the complexes in the red box lost heme binding at the time given.

**Figure 4:**
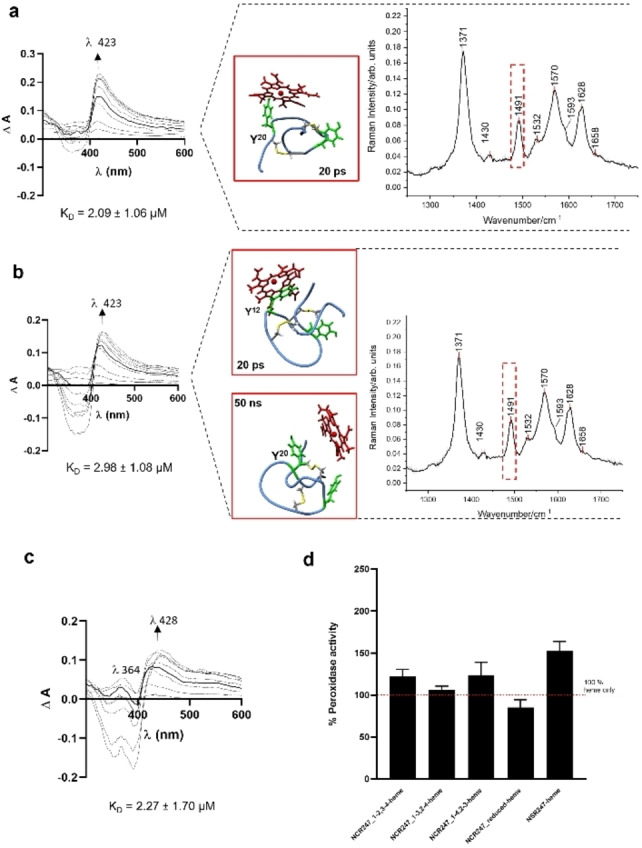
Heme‐binding analysis of NCR247 peptides. UV/vis spectroscopic analysis upon addition of increasing concentrations of heme (0.4–40 μM) to NCR247 oxidized isomers (on the left), *in silico* docking analysis (in the middle) showing residues involved in heme binding and rRaman spectra of heme‐peptide (1 : 1) complex (on the right). a, NCR247_1–3,2–4. b, NCR247_1–2,3–4. c, NCR247_reduced form, with free Cys residues. The complex formation was analysed after incubating it for 30 min at room temperature. The change in the spectral behaviour when measured after 1 min and 30 min of complex incubation is noteworthy. The intensity of the bands at λ =364 nm, indicate a change in the peptide and can be attributed to concomitant oxidation of free Cys residues in aqueous buffer systems, making the Soret band at λ =428 nm more prominent, similar to the one observed in case of the oxidized isomers. d, Peroxidase‐like activity assay of heme‐NCR247 complex measured using an oxidative conversion of TMB substrate at 652 nm.

Heme binding to the NCR247 parent peptide and its analogues was assessed using UV/vis spectroscopy (Table S2) following an earlier established experimental set‐up,[^27^, ^30^, ^31^, ^32^] with heme (0.4 to 40 μM) titrated to NCR247 peptide (20 μM) in 100 mM HEPES buffer (pH 7.0). Among the five peptides studied (Figure 1a), the reduced NCR247 peptide showed the highest heme‐binding affinity, with a K_D_ value of 1.44±0.69 μM at λ ~428 nm and representative for one heme‐binding site. The K_D_ value for this peptide was first determined by adjusting the established protocol^[30]^ to 1 min incubation time, while at an incubation time of 30 min concomitant oxidation of the cysteine residues has taken place as indicated by the fact that a stable complex was formed only in the first minutes and then a change in the spectrum occurred which is due to disulfide bond formation changing the heme‐binding behaviour of the peptide. The binding affinity evaluated at λ ~428 nm after 30 min can be attributed to the Tyr residues in the sequence and was determined to be 2.27±1.70 μM. The oxidized isomers NCR247_1–2,3–4, NCR247_1–3,2–4, and NCR247_1–4,2–3 exhibited low to moderate heme‐binding affinity in the μM range (2.98±1.08 μM, 2.09±1.06 μM, and 2.8±1.07 μM, respectively) at a Soret band shift to λ ~420 nm (Figure 3,4). In case of the serine mutant NSR247, the heme‐binding affinity is completely lost as can be seen in Figure 3c. This indicates that the reduced Cys side chains (reduced NCR27) are preferred for binding of heme, whereas in the disulfide‐bonded peptides, binding can only occur to tyrosine if it is accessible and correctly positioned with respect to the conformational impact on the sequence in case of the oxidized cysteines.

### Resonance Raman Spectroscopy of Heme‐Peptide Complexes

To explore the structural interactions between the NCR247 peptides and heme, we performed resonance Raman (rRaman) spectroscopy using the heme‐peptide (1 : 1) complexes of NCR247_reduced, the oxidized isomers and the serine mutant. The ν3 band, which is evident at 1490 cm^−1^, is present in unbound heme and pentacoordinated (5c) heme‐peptide complexes as reported earlier for Cys‐, His‐, and Tyr‐containing heme‐binding peptides and proteins.[^7^, ^10^, ^27^, ^29^] The analysis of the rRaman spectra of the NCR247‐derived peptides (Table S2) revealed heme binding in a pentacoordinated fashion, which is due to the presence of the Tyr residues in the peptides.[^30^, ^32^] The pentacoordination observed in NSR247 can be attributed to the higher concentration of heme (400 μM) employed for technical reasons in the rRaman studies, in contrast to the UV/vis experiments, which had a tenfold lower heme concentration (40 μM) at maximum.

### Peroxidase‐like activity of NCR247 Peptides in Complex with Heme

Heme exhibits an intrinsic peroxidase‐like activity[^33^, ^34^, ^35^, ^36^, ^37^] that can be increased in complex with peptides or proteins, however, is critically discussed because of the possible formation of cytotoxic reactive oxygen species (ROS).^[38]^ The NCR247‐derived peptides were tested for their peroxidase‐like activity in complex with heme (1 : 1) using a 3,3’,5,5’‐tetramethylbenzidine (TMB)‐based system.^[34]^ The % peroxidase‐like activity was normalized against the activity of heme only (100 %). All the NCR247 peptides exhibited an activity in the range of 85 to 150 % and thus did not show a noteworthy increase of the peroxidase‐like activity (Figure 4d, S4) if compared to previously studied heme‐peptide complexes.[^27^, ^32^]

### *In Silico* Analysis Of NCR247‐Derived Peptides Substantiate UV/Vis Spectroscopic Results

**Figure 5:**
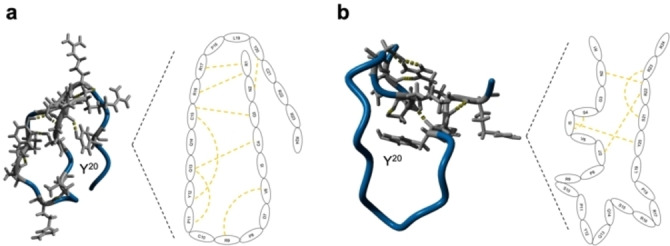
Hydrogen bonding pattern (yellow lines) for heme binding to a, the serine mutant NSR247, and b, the reduced NCR247 peptide.

In addition to the CD conformational study, the best computational model obtained from C−I‐TASSER^[39]^ was used to generate the disulfide‐bonded isomers of NCR247 and its Ser mutant (Table S3). These models were subjected to a 100 ns Molecular Dynamics (MD) simulation. From the RMSD trajectories it was obvious that isomer NCR247_1–4,2–3 was the most stable peptide, while the reduced parent peptide (NCR247_reduced) showed the highest fluctuations throughout the simulation time with RMSD as high as 15 Å during the initial stabilisation phase. It later was stabilized showing a RMSD with fluctuations of ∼ 1 Å after 60 ns (Fig. S1a). The other two isomers (NCR247_1–2,3–4 and NCR247_1–3,2–4) as well as NSR247 showed RMSD fluctuation of <1 Å after 50 ns and hence were ascertained to be stable (Fig. S1a). A stable pose was selected from the production phase (phase during which RMSD is stable along with the other parameters^[40]^ of the MD trajectory) for the docking studies (Fig. S1a). All the potential heme‐binding sites (i. e., Cys4, Cys10, Tyr12, Cys15, Tyr20, Cys21) of the NCR247 sequence were tested using a global and a focused docking approach as earlier established.[^10^, ^36^, ^41^] Binding of heme was only observed to the tyrosine residues at positions 12 and 20 in all linear and cyclic peptides, yet to a different extent The best docking poses of each heme‐peptide complex was subjected to a short MD simulation of 20 ps. In case the heme‐binding event was observed and stable within 20 ps, the complex was further simulated for additional 50 ns (Figure 3). Interestingly, out of all the complexes only NCR247_reduced and NCR247_1–4,2–3 showed a stable binding during the 50 ns simulations. The distance between the hydroxyl oxygen atom of Tyr20 and the iron ion of heme was 2.3 Å for NCR247_reduced and 2.7 Å for NCR247_1–4,2–3. To further analyse the heme binding to these peptides, the complexes were simulated for 500 ns (Figure 3a, Figure S1b). Heme lost binding to the oxidized NCR247_1–4,2–3 isoform after 176 ns (Figure 3b, Fig. S1c, Supplementary Video 1). On the contrary, NCR247_reduced showed a stable heme binding throughout the 500 ns simulation and also maintained the distance of 2.42 Å (Figure 3a, Figure S1d and Supplementary Video 2). We identified the presence of cationic‐π and π‐π interactions between the heme molecule and the Tyr20 residue, which in turn contribute to the stabilization of this association (Figure 3a). Due to the fact that NSR247 and NCR247_reduced differ solely by the presence of cysteine and serine residues and considering that cysteines in their reduced state do not participate in disulfide bridge formation, it is noteworthy that heme interacts with Tyr20 of NCR247_reduced, whereas it does not bind to this residue of NSR247 mutant. The underlying rationale for this observation was elucidated through the visualis ation of the prospective hydrogen‐bond network using YASARA (version 22.9). It was demonstrated that the oxygen atom of Tyr20 in NSR247 engages in hydrogen bonding with Asn2 (Figure 5a), thereby precluding heme binding in all docking trials conducted. Conversely, this phenomenon does not manifest in the NCR247_reduced peptide. Despite the presence of an H‐bond between Tyr20 and Ile5, the oxygen atom within the backbone participates in this hydrogen bonding, while the coordinating hydroxy group of the tyrosine residue does not engage in hydrogen bonding interactions as illustrated in Figure 5b. Thus, the reduced NCR247 is more favourable for heme binding compared to the other analogs according to the *in silico* studies.

### Maltose‐Binding Protein (MBP) Exhibits Weak Heme‐Binding Affinity

**Figure 6:**
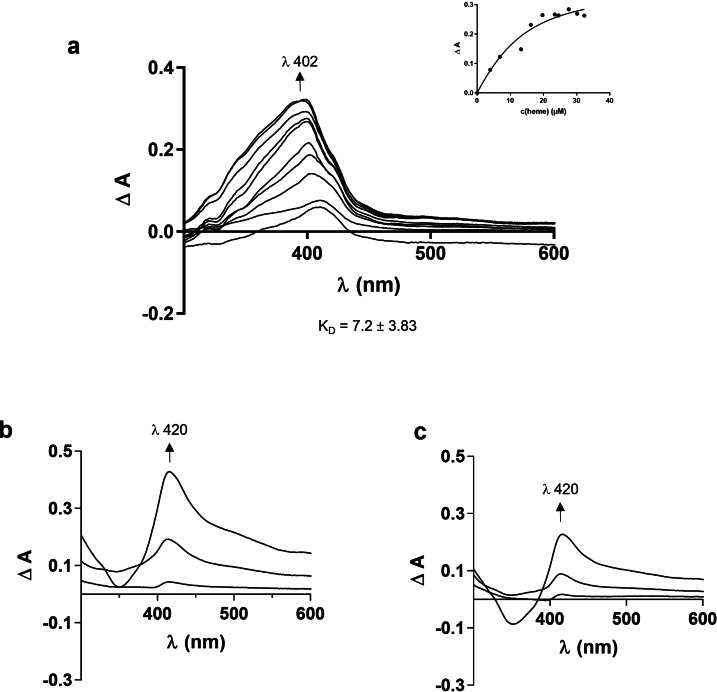
Analysis of heme‐binding to MBP and MBP‐NCR247 complexes. a, UV/vis spectroscopic evaluation of MBP (10 μM) titrated with increasing concentrations of heme. The K_D_ value reflects moderate heme binding affinity. The best‐fitting saturation curve is indicated on the right. b, UV/vis spectrum of NCR247 (10 μM) + heme (4, 20, 40 μM) further analyzed in complex with MBP (10 μM). c, UV/vis spectrum of MBP (10 μM) + heme (4, 20, 40 μM) further analyzed in complex with NCR247 (10 μM).

In order to demonstrate heme binding to the protein MBP that is part of the hexamer structure of the MBP‐NCR247 construct earlier suggested to possess a high heme‐binding capacity^[11]^, we started by identifying putative heme‐binding motifs (HBMs) in MBP using HeMoQuest.^[42]^ Two potential Tyr‐based HBMs, i. e., YAFKY^172^ENGK and SAFWY^342^AVRT (Table S4), were suggested. We thus conducted UV/vis measurements[^36^, ^41^] by titrating increasing concentrations of heme (4–40 μM) to the protein (10 μM). The resulting complex exhibited a Soret band shift to λ ∼400 nm with increasing intensity proving heme binding to MBP (Figure 6a). The binding affinity,[^30^, ^43^] however, was determined to be only moderate with a K_D_ value of 7.20±3.83 μM for a stoichiometry of 1 : 1 (MBP:heme).

**Figure 7:**
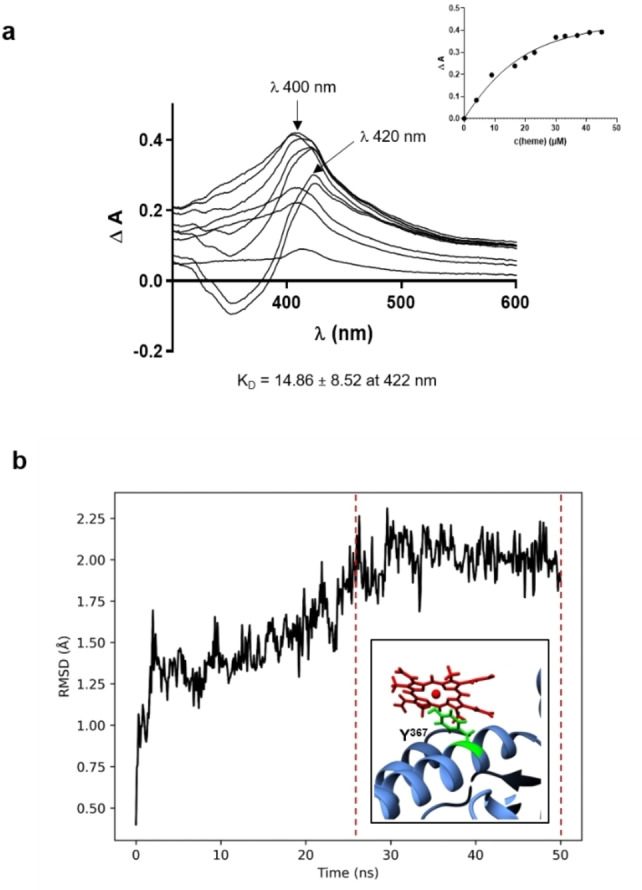
Analysis of heme binding to MBP and the MBP‐NCR247 complex. a, UV/vis spectroscopic analysis upon addition of increasing concentrations of heme (4–45 μM) to NCR247_oxidized (1–4,2–3) isomer in complex with MBP. b, RMSD of MBP (PDB: 7MQ7) in complex with heme at Tyr367, simulated for 50 ns with stable phase highlighted with red dotted lines.

To gain further insight into the binding behaviour of MBP with heme in combination with NCR247 derivatives, other UV/vis titration approaches were carried out, adapted from earlier reported protocols.[^36^, ^41^] Interestingly, heme binding to MBP (10 μM) in complex with NCR247_1–4,2–3 (10 μM) after 30 min incubation time exhibited a shift in the Soret band of heme to ∼400 nm at lower concentrations (up to 25 μM) of heme and further to λ ∼420 nm at higher concentrations (30–45 μM) (Figure 7a). Thereby, a K_D_ value of 14.86±8.52 μM with a stoichiometry of 1 : 1 (MBP‐NCR247_1–4,2–3:heme) was found at λ ∼420 nm, using the best fit model[^30^, ^43^] For a higher stoichiometry, i. e., two heme molecules binding to the MBP‐NCR247_1–4,2–3 complex, a lower K_D_ value of 7.6±2.4 μM was observed, indicating a higher heme‐binding affinity. The profile of these spectra reflects more the NCR247_1–4,2–3‐heme interaction (Figure 3b), which may indicate that heme binding to the NCR247 peptide is now occurring in addition to heme association with MBP in a mixture of both, NCR247 and MBP, at higher heme concentrations. Such a binding scenario, however, would rather suggest a 6 : 12‐mer complex formation compared to the 6 : 6 complex with heme only binding to NCR247 as hypothesized in the earlier report,^[12]^ yet only in case the hexamer structure in indeed formed.

Additionally, NCR247_1–4,2–3 (10 μM) was incubated with heme (4, 20, 40 μM) for 30 min and then 10 μM MBP was added to the heme‐NCR247_1–4,2–3 complex and incubated for another 30 min (Figure 6b). The resulting spectrum revealed the same profile as already discussed for the NCR247_1–4,2–3‐heme complex (Figure 3b). Similarly, MBP (10 μM) was first incubated with heme (4, 20, 40 μM) for 30 min followed by the addition of 10 μM of NCR247_1–4,2–3 and incubation of the mixture for further 30 min (Figure 6c). Again, this mixture showed an NCR247‐like heme‐binding behaviour during the spectroscopic measurements. Both experiments resulted in the same outcome as aforementioned for the incubation of heme with the preformed MBP‐NCR247_1–4,2–3 complex, i. e., the NCR247 peptide preferentially binds heme even in the presence of MBP, which can be attributed to the two‐fold higher binding affinity of the peptide compared to the protein.

The global docking studies demonstrated that the potential heme‐binding site in MBP is Tyr342 because the largest cluster of potential heme‐binding conformations were formed around this residue. The extracted pose with 3.59 Å between the heme iron and the tyrosine side chain was subjected to a 50 ns MD simulation. During the simulation, the distance reduced to 2.16 Å and the complex got further stabilized as can be seen in the RMSD plot (Figure S1).

## Discussion

Given the fact that targeting heme‐uptake processes in bacterial infections is a viable approach to combat diseases, huge efforts are undertaken to identify novel strategies by e. g., exploiting natural product resources from plants, vertebrates and invertebrates, respectively.[^44^, ^45^, ^46^] The earlier reported suggestion to interfere with the accessibility of heme for pathogenic bacteria by employing higher order heme‐NCR247 peptide aggregates[^11^, ^47^] seemed to be a valuable approach in this regard. Although the NCR247 peptides have been shown to possess favourable properties concerning cytotoxicity in melanoma cells and hemolysis in human erythrocytes,^[20]^ research towards the heme‐binding capacity and consequently therapeutic potential for heme sequestration of these antimicrobial peptides requires verification. Leveraging the vast knowledge of transient heme‐binding interactions[^24^, ^28^, ^31^, ^36^, ^42^, ^48^, ^49^, ^50^, ^51^, ^52^] with peptides and proteins established in the past, we established an effective strategy to evaluate the heme‐binding properties and binding modes by various spectroscopic methods, structural and *in silico* studies.

**Figure 8:**
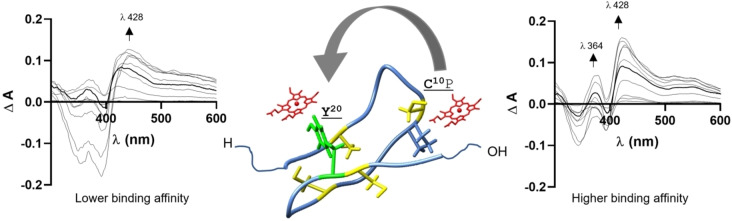
Suggested transfer mechanism for heme binding to Cys10 and Tyr20 of the reduced NCR247 peptide. The complex formed after 1 min shows a distinct spectroscopic pattern characteristic for a CP‐based motif (UV group 3)7 with high heme‐binding affinity (spectrum on the right). The nature of the complex (spectrum on the left), however, changes over a period of 30 min incubation time, due to concomitant oxidation of the free Cys residues in the peptide, including the CP‐motif. The spectrum obtained is characteristic for Tyr‐based motifs (UV group 1).[^7^, ^32^]

The heme‐binding experiments of NCR247_reduced possessing free cysteine residues for heme coordination, the standard incubation time of 30 min for the formation of the heme‐peptide complex significantly altered the UV/vis spectrum indicating a change in the peptide's conformation compared to the spectrum received after 1 min (Figure 4c). The spectrum from the longer incubation time reflects the one observed for the oxidized isomers (Figure 3a, 8), thus indicating the oxidation of the cysteine residues to disulfide bonds and consequently a change in the coordination sphere of the heme iron from a cysteine thiolate to a hydroxyl group of a tyrosine side chain (Figure 5).[^28^, ^32^] Therefore, our observations for the reduced version of the 24mer NCR247 and its three oxidized disulfide isomers are not in agreement with the spectra reported by Sankari *et al*.^[11]^ Indeed, none of our earlier studied Cys‐, His‐, and Tyr‐based peptides with either one or two coordination sites of the length of 9–40 amino acids showed a UV/vis spectrum possessing a maximum at 450 nm as found in the aforementioned report.^[11]^ Instead, a Soret band shift to 450 nm may be indicative of cysteine as an axial ligand to the heme iron as has been shown e. g., for Cytochrome P450.[^53^, ^54^]

In addition to the shape of the UV/vis spectra for all the peptides investigated herein, we were not able to reproduce the heme‐binding affinities described earlier.^[11]^ For example, the best binding affinity for our series of peptides (Tables S1, S2) was found for the reduced version (1.44±0.69 μM at 1 min of incubation time), which, however, is not stable under non‐inert conditions. It is more likely that the binding affinities of the oxidized disulfide isomers are representative of the actual heme‐binding efficiency of NCR247 in the range of 2–3 μM (Figure 3b, Table S2) and thus, the nM affinity given by Sankari *et al*. could not be confirmed by our studies. Due to the absence of heme binding to the serine mutant NSR247 (Figure 3c, 5), it is also not convincible to reproduce the analogues described earlier, such as the C10P11SA, the C10P11SA/C15S or the Y12 A mutant,^[11]^ which were also suggested to possess nM affinity, however, that is not justified by our results.

In the previous study by Sankari *et al*., a hexamer structure of the MBP‐NCR247 construct was suggested to bind six heme molecules. Since detailed sequence information, especially concerning the oxidation state of NCR247 in the construct, has not been provided in their report, further UV/vis titration experiments with the two components of the construct were carried out. This was based on the idea that one major contribution to the heme‐binding affinity for transient heme association to peptides and proteins results from the coordinating residue and partially the other from the surrounding amino acids. However, a HeMoQuest^[42]^ search for potential heme‐binding motifs in MBP revealed no prominent HBM that may be able to confer strong heme binding to the protein. Thus, to gain a deeper insight into the binding behaviour of MBP in combination with NCR247 derivatives we selected NCR247_1–4,2–3 in a combination experiment with three components in the mixture, i. e., MBP, NC247 peptide, and heme, which were sequentially added in different order to identify binding preferences and potential transfer options for heme to either MBP or NCR247.

Subsequently, it was observed that at lower heme concentrations (up to 25 μM), heme is not accessible to bind NCR247, but possibly becomes available at higher concentrations (30–45 μM). Thus, structural evidence is crucial to support this theory. The two peaks in the Soret region, observed for the binding event of MBP+NCR247 with heme show a co‐existing equilibrium of a mixture of complexes formed, that is MBP‐heme, NCR247‐heme, MBP+NCR247‐heme (Figure 6a). However, NCR247 is clearly not a strong heme‐binder and the affinity in sub‐nM range for the reduced form and in μM range for the serine mutant, is over interpreted in the aforementioned study.^[11]^ Moreover, it is not to be classified as a non‐binder, which could have been predicted beforehand according to the sequences of the molecules. Taken together, the coordination‐sensitive ν3‐vibrational band caused by Cα−Cβ stretching vibration observed around 1490 cm^−1^ for all heme‐NCR247 peptide complexes, except NCR247_reduced form, in the rRaman spectra as well as the pentacoordination observed the *in silico* analysis, highlight the substantial contribution of Tyr residues in the peptide to the heme‐binding behaviour (Figure 3). Conferring to our results, MBP itself is binding heme with a moderate‐low affinity (Figure 6a). Therefore, a moderate binding can be due to a cumulative effect of both, weak NCR247 and weak MBP binding.

A further hint of the moderate heme binding capacity and less strong binding affinity comes from the peroxidase‐like activity^[32]^ of the complexes formed. However, the NCR247 peptide and its derivatives from the present study did not show a significant effect on peroxidase‐like activity of heme (Figure 4d), in contrast to formerly published heme‐binding peptides.[^27^, ^32^] Previous studies have also stated that the peptides containing less basic amino acids along with other sequence specificities render them to possess a significant peroxidase‐like activity.^[32]^ Attributing to the presence of seven Arg residues, it is thus evident that the NCR247‐derived peptides did not show a significant increase in the peroxidase‐like activity.

In conclusion, a detailed examination of the structural aspects of such Cys‐rich and multiple disulfide‐bridged peptides with respect to heme binding is essential in order to understand the intricate interplay of the binding events as this is a prerequisite to assess how heme binding can affect the function of such plant‐peptides in the context of host‐bacteria symbiosis or such naturally occurring peptides can serve as lead structures for drug design and development.

## Author Contributions

D.I. conceptualized and designed the project. S.M.V. performed the peptide synthesis, analysis, and UV experiments. A.R. and U.N. designed, performed and analysed the Raman experiments. D.C.R. carried out the computational studies. S.M.V., D.C.R., A.R., U.N., and D.I. analysed the data. The manuscript was written by S.M.V., D.C.R., and D.I, and finally approved by all authors.

## Conflict of Interests

The authors declare no conflict of interest.

1

## Supporting information

As a service to our authors and readers, this journal provides supporting information supplied by the authors. Such materials are peer reviewed and may be re‐organized for online delivery, but are not copy‐edited or typeset. Technical support issues arising from supporting information (other than missing files) should be addressed to the authors.

Supporting Information

Supporting Information

Supporting Information

## Data Availability

The data that support the findings of this study are available from the corresponding author upon reasonable request.
