# Supplementary material for: Molecular Insights into the Heme‐Binding Potential of Plant NCR247‐Derived Peptides
Source: Chembiochem. 2025 Jan 20;26(5):e202400920. doi: 10.1002/cbic.202400920 (PMC11875561; doi:10.1002/cbic.202400920)
Supplement: Supplementary file 1 — Supporting Information [file CBIC-26-e202400920-s003.pdf]

ChemBioChem

Supporting Information

# Molecular Insights into the Heme-Binding Potential of Plant NCR247-Derived Peptides

Sonali M. Vaidya, Dhruv C. Rathod, Anuradha Ramoji, Ute Neugebauer, and Diana Imhof*

ChemBioChem

# Supporting Information

## Molecular Insights into the Heme-binding Potential of Plant NCR247-derived Peptides

Sonali M. Vaidya [a], Dhruv C. Rathod [a], Anuradha Ramoji [b][c], Ute Neugebauer [b][c][d] Diana Imhof*[a].

[a] Sonali M. Vaidya, Dhruv C. Rathod, Prof. Dr. Diana Imhof
Department: Pharmaceutical Biochemistry and Bioanalytics
Institution: Pharmaceutical Institute, University of Bonn
Address 1: An der Immenburg 4, Bonn, Germany.
E-mail: dimhof@uni-bonn.de

[b] Dr. Anuradha Ramoji, Prof. Dr. Ute Neugebauer
Department: Leibniz Institute of Photonic Technology
Institution: Member of Leibniz Health Technologies, Member of the Leibniz Centre for Photonics in Infection Research (LPI)
Address 2: Jena, Germany.

[c] Dr. Anuradha Ramoji, Prof. Dr. Ute Neugebauer
Department: Institute of Physical Chemistry (IPC) and Abbe Center of Photonics (ACP)
Institution: Member of the Leibniz Centre for Photonics in Infection Research (LPI), Friedrich-Schiller-University Jena.
Address 3: Jena, Germany.

[d] Prof. Dr. Ute Neugebauer
Department: 4Center for Sepsis Control and Care
Institution: Jena University Hospital.
Address 4: Jena, Germany.

## Methods.

**Synthesis and analytical characterization of NCR247 peptides.**

The NCR247 peptides and derivatives including the all-serine mutant (control) were synthesized by automated solid-phase peptide synthesis using an EPS221 peptide synthesizer (Intavis Bioanalytical Instruments AG, Cologne, Germany). After assembly, the crude peptides were purified by semipreparative reversed-phase high performance liquid chromatography (RP-HPLC) on a LC-8A system (Shimadzu, Duisburg, Germany) equipped with a Knauer C18 Eurospher 100-5 column, as described previously.[1–3] The pure fractions were collected and characterized by analytical RP-HPLC on a LC-20A system (Shimadzu, Duisburg, Germany) equipped with either a C18 (Vydac 218TP54 column; 250 × 4.6 mm, 5 µm particle size, 300 Å pore size) or a C8 column (Vydac 208TP54 column; 250 × 4.6 mm, 5 µm particle size, 300 Å pore size). The elution was performed with a gradient of 10−40% eluent B in 30 min. The flow rate was 1 mL/min with eluent A: 0.1% TFA in water and eluent B: 0.1% TFA in acetonitrile. The detection of the peptides was at λ = 220 nm. For characterization of molar masses of the peptides, matrix-assisted laser desorption mass spectrometry MALDI-TOF-MS was employed and the spectra were measured on an ultrafleXtrem TOF (Bruker Daltonics GmbH, Bremen, Germany). The chromatography and MS results are summarized in Table S1.

**Oxidation of cysteines in NCR247_1-2,3-4, NCR247_1-3,2-4, and NCR247_1-4,2-3.**

Under oxidative reaction conditions, the cysteines were deprotected and folded by formation of the disulfide bridges between the cysteines according to protocols reported earlier.[4,5] Thereby, the disulfide bridge between the free cysteines, obtained from the cleavage of the Trt groups during peptide removal from the resin forms first, then the Acm protecting groups are removed in solution by adding iodine (stock solution: 0.1 M in methanol) to the peptide solution and the second disulfide bridge is formed by subsequent addition of 10 equivalence of iodine, in situ.[5,6] Briefly, 20 mg of the respective NCR247_peptide (Mw = 3151.68 g) was dissolved in 50% glacial acetic acid to obtain a final peptide concentration of 0.05 mM.. The oxidation reaction is started first by the addition of 1.1 equivalence of iodine under argon environment. After one hour, an additional 8.9 equivalence of iodine is added and the mixture is stirred for further 4 hours. The aqueous reaction mixture is extracted three times using the same volume of ethyl acetate. The aqueous solutions containing peptides is combined, freeze-dried, and stored at -20 °C. Reaction controls were taken at regular time intervals during the oxidation. Samples were taken immediately after the start of oxidation and then at each subsequent hour. The aqueous phase is subsequently analysed using the analytical Shimadzu LC-20A system. The oxidized isomers are purified by semipreparative RP-HPLC on a LC-8A system as described above.[1,2] The pure fractions are collected and characterized by analytical RP-HPLC using two systems and MALDI-TOF-MS as described above. To confirm the disulfide bridge formation, carbamidomethylation using 4 mM iodacetamide in 10 mM phosphate buffer (pH 7.8) was carried out for 2 hours at room temperature before analysis by MALDI-TOF-MS.

**Preparation of heme stock solution.**

If not stated otherwise, Fe(III)PPIX (hemin, herein referred to as “heme”) from Frontier Scientific (Logan, UT, USA) was dissolved in 30 mM NaOH to a final concentration of 1 mM, as described previously,[2] was used as the stock solution and freshly prepared prior to immediate use. The solution was further diluted in the buffer system and concentration as required for the respective experiment.

**Analysis of heme binding to NCR247-derived peptides by UV/vis spectroscopy.**

Heme binding to the NCR247-derived peptides (Fig. 1, Table S1) was investigated by UV/vis spectroscopy, as described earlier.[2,3,7–9] In brief, the peptides (10 or 20 µM) were incubated for 30 min or 1 min (in case of NCR247_reduced) with varying concentrations of heme (0.4 - 40 µM) in 100 mM HEPES buffer (pH 7.0). Absorbance spectra were recorded on a Multiskan GO spectrophotometer (ThermoScientific, Dreieich, Germany) in the range of 300 - 600 nm. Difference spectra were generated by subtracting the spectra of heme and peptide alone from the absorbance spectrum of the hemin-peptide complex. Dissociation constants ($K_D$) were determined by using GraphPad Prism 10.0.2 and the earlier established equation from Pîrnău and Bogdan, as described elsewhere.[7,10]

**Peroxidase-like activity of the heme-peptide complexes.**

Determination of the peroxidase-like activity of the NCR247-derived peptides in complex with heme was performed as reported.[11,12] Thereby, 42 µM of heme in (buffer, pH 7.4) was preincubated with an equimolar solution of the respective peptide or protein in 0.1 M phosphate buffered saline (PBS) buffer (pH 7.4) for 30 min. A mixture of 3,3',5,5'-tetramethylbenzidine (TMB; 1.66 mM) in 0.12 M HCl and $H_2O_2$ (163.2 mM) in 0.1 M citrate buffer (pH 5.0) served as the substrate.[11] 10 µl of the heme-peptide complex (final concentration: 1 µM) were added to 200 µl of the substrate. The absorbance was recorded at 652 nm as kinetic measurement performed over a period of 10 min to monitor the oxidation of the substrate. Data were normalized against the peroxidase-like activity of heme only (100%) and evaluated as the average of triplicate measurements.

**UV/vis titration experiments of peptides and proteins with heme.**

Maltose-binding protein (Rockland Inc. Pennsylvania, USA) (10 µM) was preincubated with heme in different concentrations (4 - 40 µM) for 30 min in HEPES buffer (pH 7.0) prior to the UV/vis spectroscopic analysis. Subsequently, absorbance spectra were recorded and analysed analogously to the heme-binding studies with the peptides described above.[2] Similar to the aforementioned approach, 10 µM MBP was incubated with increasing concentrations of heme (4 - 40 µM) in HEPES buffer (pH 7.0) for 30 min. Then, 10 µM of NCR247 was added and measured spectroscopically. In addition to this, 10 µM NCR247_1-4,2-3 was incubated with increasing concentrations of heme (4 - 40 µM) in HEPES buffer (pH 7.0) and 10 µM MBP was added to evaluate the heme-binding interaction.

**Resonance Raman spectroscopy of the heme-peptide complexes.**

Resonance Raman spectroscopic measurements were conducted using a micro-Raman setup (CRM 300, WITec GmbH, Germany), which was equipped with a TopMode-405nm diode laser excitation wavelength (Toptica Photonics AG, Germany). The Raman system is connected to an upright microscope with a motorised XY microscope stage and a 10x/0.2 NA objective (Zeiss GmbH, Germany), which serves to focus the incoming laser light onto the sample as well as for the collection of the 180° backscattered light. A 100 µm optical fibre was employed to guide the Raman signal into the spectrometer, with a back-illuminated CCD (DV401A-BV-352 cooled to -60°C (ANDOR, Ireland, 1024x127 pixels) used for detection. An aqueous solution of heme (400 µM) in 1x PBS, pH 7.0, and peptide were combined in an equimolar ratio. The reaction mixture was incubated for 30 minutes at room temperature and subsequently centrifuged in order to remove any precipitate, prior to measurement of the resulting supernatant solution. The samples and control solutions were measured in an in-house manufactured open quartz glass cuvette mounted on a motorised holder, which was rotated spirally at a speed of 1 rotation per 60 seconds. Raman spectra were collected with an integration time of 30 seconds at a laser power of 22 mW. Each measurement consisted of five spectra per sample at a spectral centre of 1100 $cm^{-1}$ with a grating of 1800 lines/mm, in order to obtain a high spectral resolution and thereby reveal minimal changes in the band positions. The raw Raman spectra were processed using the open-source software GNU R (R Core Team, 2021) and an in-house algorithm, and the resulting data were visualised using the graphing software OriginPro 2016G Sr2. The Raman spectra were preprocessed, including the removal of cosmic spikes, the selection of the wavenumber region (300–2200 $cm^{-1}$), background correction using a sensitive non-linear iterative peak-clipping (SNIP) algorithm, and vector normalisation. The mean spectra were calculated as the average of five measurements.

**Circular Dichroism (CD) spectroscopy with NCR247-derived peptides.**

At a constant temperature of 20 °C, CD spectroscopy measurements were carried out on a JASCO J-715 spectropolarimeter. The experimental set-up was adapted to an earlier described protocol[7,8] The spectra were recorded from 180 to 260 nm with a scan speed of 100 nm*$min^{-1}$, a band width of 1 nm, an accumulation of 5 scans, and a resolution of 0.20 nm using a 1 mm quartz cuvette. Baseline correction was performed using the spectrum of a 50 mM sodium phosphate buffer (pH 7.4). All the peptide solutions were prepared in the same buffer. The ellipticity of the CD spectra was expressed in millidegrees and later converted to molar ellipticity (θ) in deg*$cm^2$*$dmol^{-1}$. The spectra were evaluated and presented with GraphPad Prism 10.0.2.

***In silico* studies of NCR247-derived peptides and their respective heme-complexes.**

The peptide sequence of NCR247 [RNGCIVDPRCPYQQCRRPLYCRRR] was given as an input to the online C-I-TASSER structure prediction tool. The suggested best model was selected for the further *in silico* analysis by YASARA (version 22.9). The structure generated from C-I-TASSER did not contain any disulfide bridge and was thus considered the reduced version of NCR247. In order to study the oxidized variants, the three possible isomers were generated by establishing disulfide bridges between the four available

cysteines (1-4) as follows: NCR247_1-2,3-4, NCR247_1-3,2-4 and NCR247_1-4,2-3. The serine mutant was generated by substitution of the cysteines with serine residues (NSR247). All the disulfide-bonded isomers and the serine mutant were generated with YASARA (version 22.9) prior to molecular dynamics (MD) simulations to tests their structure. Therefore, all the isomers and mutants were subjected to a 100 ns MD simulation and snapshots from the final 20 ns trajectory were selected for further analysis. Heme docking simulations were performed as described earlier by using the AutoDock Vina algorithm integrated in YASARA (version 22.9). In brief, a cubic simulation cell (10 Å × 10 Å × 10 Å) was built around the heme-coordinating residue for the HBMs of interest. A successful heme docking to a peptide was defined as pose where the distance between the $Fe^{3+}$ ion of heme and the respective non-protonated nitrogen atom of histidine or the hydroxyl oxygen of tyrosine residue was ≤ 3 Å. Subsequently, the best docking poses were subjected to a 100 ns MD simulation to verify the stability of heme binding at the docked sites and was further extended to 500 ns for the stability checks. Additional docking studies were performed to analyse the putative docking regions of NCR247 and heme to MBP (PDB ID 7MQ7). At first, a simulation box was built around the whole protein and global docking was performed with NCR247 as a ligand, then the global docking of heme to the MBP-NCR247 complex was performed.

**Supplementary Table S1:** Analytical characterization of NCR247 peptides

| Peptide | Sequence | HPLC $t_R$ [min] [a] | HPLC $t_R$ [min] [b] | $M_W$ (theor.)[c] | $M_W$[d] |
|---|---|---|---|---|---|
| NCR247_reduced | RNG**C**IVDPR**C**PYQQ**C**RRPLY**C**RRR | 17.95 | 17.81 | 3003.47 | 3003.12 |
| NCR247_1-2,3-4 | RNG**C**IVDPR**C**PYQQ**C**RRPLY**C**RRR | 19.93 | 20.52 | 3007.57 | 3007.30 |
| NCR247_1-4,2-3 | RNG**C**IVDPR**C**PYQQ**C**RRPLY**C**RRR | 17.89 | 18.86 | 3007.57 | 3007.30 |
| NCR247_1-3,2-4 | RNG**C**IVDPR**C**PYQQ**C**RRPLY**C**RRR | 17.81 | 17.51 | 3007.57 | 3007.30 |
| NSR247 | RNG**S**IVDPR**S**PYQQ**S**RRPLY**S**RRR | 16.78 | 17.02 | 2943.56 | 2945.3 |

The retention times are stated in minutes as observed by analytical HPLC on [a]RP-C18 Knauer Eurospher column, [b]RP-C8 Vydac column, eluent A: water with 0.1 % TFA, eluent B: acetonitrile with 0.1 % TFA, 10%-40% eluent B in 30 min; [c]theoretical monoisotopic mass peaks [M]; [d]monoisotopic mass peaks detected as $[M+H]^+$.

**Supplementary Table S2:** Spectroscopic analysis of heme-binding to NCR247 peptides

| | **UV/Vis spectroscopy** | | | **Raman geometry** | |
|---|---|---|---|---|---|
| **Peptide** | **$K_D$ [µM]** | **n** | **Soret band [nm]** | **$v_3$-band [$cm^{-1}$]** | **$v_2$-band [$cm^{-1}$]** |
| NCR247_reduced* | 1.44 ± 0.96 (1') #<br>2.27 ± 1.70 (30') # | 1 | 428 | -<br>1491 (5c) | -<br>1570 |
| NCR247_(1-2,3-4) | 2.98 ± 1.88 | 1 | 423 | 1494 (5c) | 1570 |
| NCR247_(1-4,2-3) | 2.8 ± 1.67 | 1 | 418 | 1494 (5c) | 1570 |
| NCR247_(1-3,2-4) | 2.09 ± 1.06 | 1 | 423 | 1494 (5c) | 1570 |
| NSR247 | n.b. | - | - | 1491 (5c) | 1570 |

# Values in brackets give incubation time in minutes; *No $K_D$ evaluation was possible for $\lambda$ = 364 nm, due to low intensity of the spectrum. n= no. of heme-binding sites considered for $K_D$ evaluation., n.b. non-binding; 5c. pentacoordination.

**Supplementary Table S3:** C-I-TASSER results of NCR247 model

| Rank[a] | Model name | C-score[b] | Estimated TM-score[c] | Estimated RMSD[c] |
|---|---|---|---|---|
| **1** | **model1** | **-2.06** | **0.47±0.15** | **5.2±3.3Å** |
| **2** | **model2** | **-2.35** | **-** | **-** |
| **3** | **model3** | **-2.57** | **-** | **-** |
| **4** | **model4** | **-5.66** | **-** | **-** |
| **5** | **model5** | **-5.66** | **-** | **-** |

[a]C-I-TASSER simulations generate a large ensemble of structural conformations, i.e. decoys. These decoys are clustered by SPICKER based on pairwise structure similarity to report up to five final models from the five largest clusters. Models are ranked in descending order of cluster size. If the simulations converge well, it is possible to have less than 5 models generated, which is usually an indication of good model quality. [b]The model confidence is quantified by C-score, calculated based on significance of threading template alignments, convergence of C-I-TASSER simulations and contact map satisfaction rate. C-score is typically in the range of [-5, 2], with higher C-score signifies higher model confidence. [c]Model and RMSD are estimated based on C-score and protein length for the first model.

**Supplementary Table S4:** HeMoQuest[13] predicted HBMs in MBP (Uniprot Accession: P0AEX9)

Input sequence:

```
MKIEEGKLVIWINGDKGYNGLAEVGKKFEKDTGIKVTVEHPDKLEEKFPQVAATGDGPDIIFWAHDRFGGYAQSGLLAEITPDKAFQDKLYPFTWDA
VRYNGKLIAYPIAVEALSLIYNKDLLPNPPKTWEEIPALDKELKAKGKSALMFNLQEPYFTWPLIAADGGYAFKYENGKYDIKDVGVDNAGAKAGLT
FLVDLIKNKHMNADTDYSIAEAAFNKGETAMTINGPWAWSNIDTSKVNYGVTVLPTFKGQPSKPFVGVLSAGINAASPNKELAKEFLENYLLTDEGL
EAVNKDKPLGAVALKSYEEELAKDPRIAATMENAQKGEIMPNIPQMSAFWYAVRTAVINAASGRQTVDEALKDAQTRITKLS
```

| Coord. residue | 9mer motif | Net charge |
|---|---|---|
| Y172 | YAFKYENGK | +1 |
| Y342 | SAFWYAVRT | +1 |

**Supplementary Figure S1.**

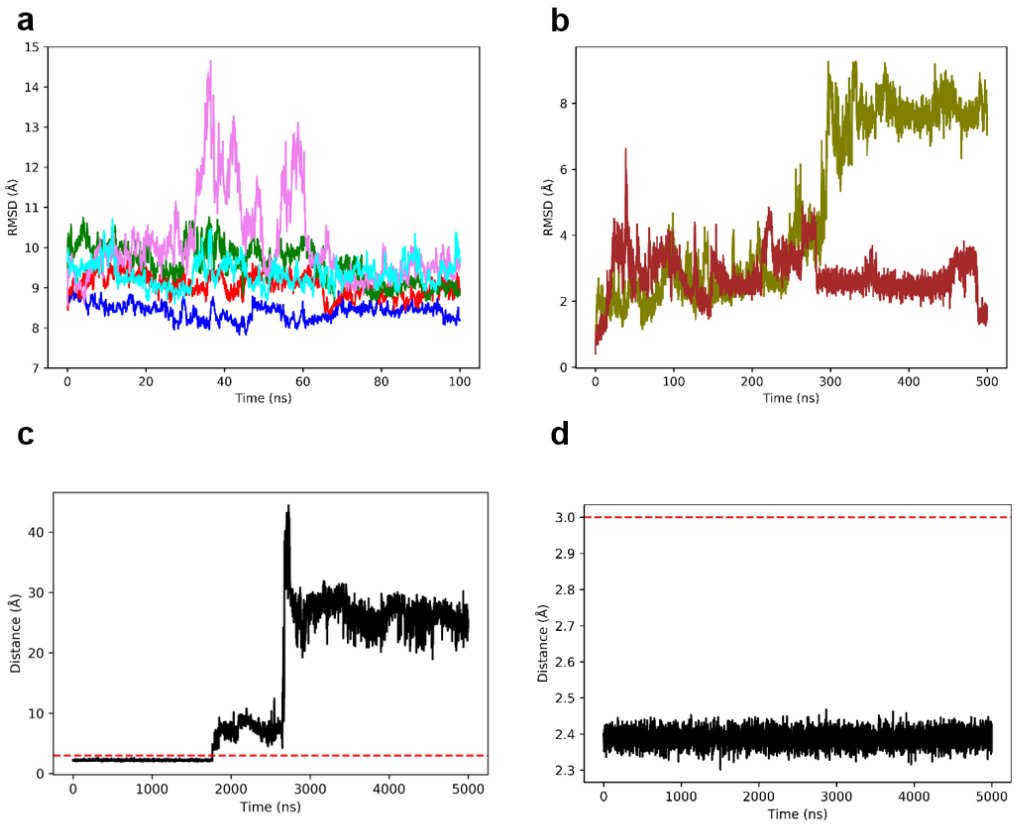


***In silico* analysis of NCR247 isoforms. a,** RMSD plots of NCR247_reduced (violet), NSR247 (cyan), NCR247_1-2,3-4 (blue), NCR247_1-3,2-4 (red), NCR247_1-4,2-3 (green). **b,** RMSD plots for NCR247_reduced (red) and NCR247_1-4,2-3 (olive) in complex with heme. **c,** Distance between 'O' of Tyr20 and "Fe" of heme in NCR247_1-4,2-3 – heme complex. **d,** Distance between 'O' of Tyr20 and "Fe" of heme in NCR247_reduced– heme complex. A red dashed line represents the minimum distance of 3 Å, which required between coordinating residue and Fe ion of heme in a heme complex.

**Supplementary Figure S2.**

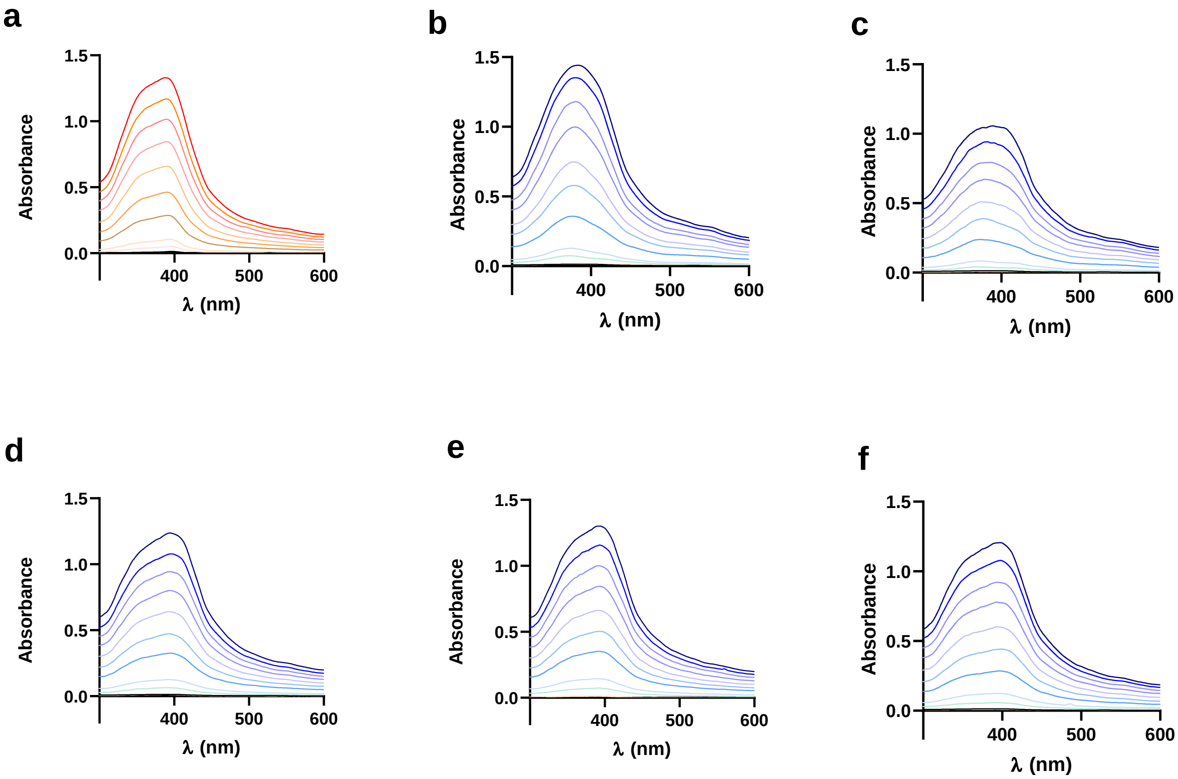


**UV/vis spectra of heme only and NC247 peptides (20 μM) in complex with heme (0.4-40 μM).** a, heme only. b, NCR247_reduced (1 min). c, NCR247_reduced (30 min). d, NCR247_1-2,3-4. e, NCR247_1-3-,2-4. f, NCR247_1-4,2-3.

**Supplementary Figure S3.**

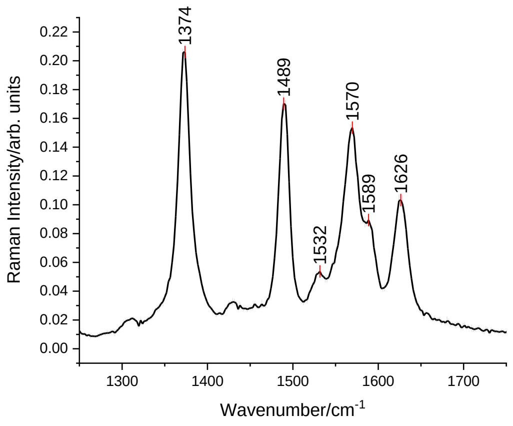


**Resonance Raman spectrum of heme ($\lambda_{ex}$ = 405 nm).**

**Supplementary Figure S4.**

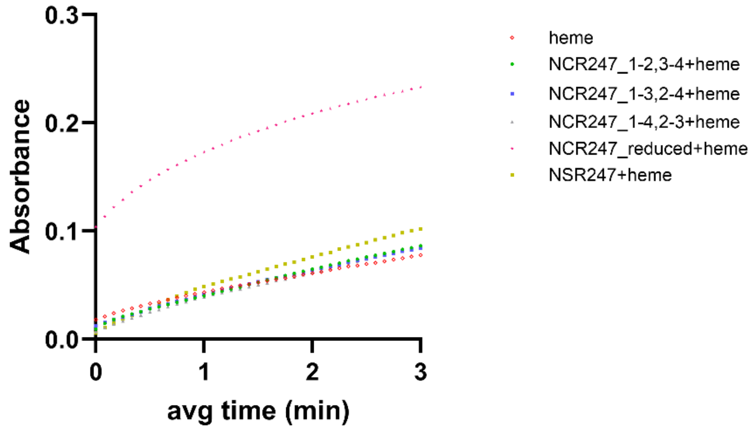


**Kinetic measurements of heme and heme-NCR247 peptide complexes.**

## References


[1] M. S. Detzel, B. F. Schmalohr, F. Steinbock, M. T. Hopp, A. Ramoji, A. A. P. George, U. Neugebauer, D. Imhof, *Biol. Chem.* **2021**, *402*, 675–691.

[2] M.-T. Hopp, D. C. Rathod, K. H. Winn, S. Ambast, D. Imhof, *Biol. Chem.* **2022**, *403*, 1055–1066.

[3] M. T. Hopp, N. Alhanafi, A. A. Paul George, N. S. Hamedani, A. Biswas, J. Oldenburg, B. Pötzsch, D. Imhof, *Antioxidants Redox Signal.* **2021**, *34*, 32–48.

[4] C. A. Bäuml, T. Schmitz, A. A. Paul George, M. Sudarsanam, K. Hardes, T. Steinmetzer, L. A. Holle, A. S. Wolberg, B. Pötzsch, J. Oldenburg, A. Biswas, D. Imhof, *J. Med. Chem.* **2019**, *62*, 3513–3523.

[5] T. Schmitz, A. Abisheck, P. George, B. Nubbemeyer, C. A. Bäuml, T. Steinmetzer, O. Ohlenschläger, A. Biswas, D. Imhof, **2021**.

[6] T. Kühl, A. Wißbrock, N. Goradia, N. Sahoo, K. Galler, U. Neugebauer, J. Popp, S. H. Heinemann, O. Ohlenschläger, D. Imhof, *ACS Chem. Biol.* **2013**, *8*, 1785–1793.

[7] T. Kühl, N. Sahoo, M. Nikolajski, B. Schlott, S. H. Heinemann, D. Imhof, *ChemBioChem* **2011**, *12*, 2846–2855.

[8] M. T. Hopp, A. A. Paul George, A. Ramoji, A. Pepanian, M. S. Detzel, U. Neugebauer, D. Imhof, *Int. J. Pept. Res. Ther.* **2022**, *28*.

[9] B. F. Syllwasschy, M. S. Beck, I. Družeta, M. T. Hopp, A. Ramoji, U. Neugebauer, S. Nozinovic, D. Menche, D. Willbold, O. Ohlenschläger, T. Kühl, D. Imhof, *Biochim. Biophys. Acta - Gen. Subj.* **2020**, *1864*.

[10] A. Pıˆrnaˇu, M. Bogdan, *Rom J Biophys* **2008**, *18*, 49–55.

[11] A. Wißbrock, N. B. Goradia, A. Kumar, A. A. Paul George, T. Kühl, P. Bellstedt, R. Ramachandran, P. Hoffmann, K. Galler, J. Popp, U. Neugebauer, K. Hampel, B. Zimmermann, S. Adam, M. Wiendl, G. Krönke, I. Hamza, S. H. Heinemann, S. Frey, A. J. Hueber, O. Ohlenschläger, D. Imhof, *Sci. Rep.* **2019**, *9*.

[12] H. Atamna, M. Brahmbhatt, W. Atamna, G. A. Shanower, J. M. Dhahbi, *Metallomics* **2015**, *7*, 309.

[13] A. A. Paul George, M. Lacerda, B. F. Syllwasschy, M. T. Hopp, A. Wißbrock, D. Imhof, *BMC Bioinformatics* **2020**, *21*.
